# Risk-stratified and stepped models of care for back pain and osteoarthritis: are we heading towards a common model?

**DOI:** 10.1097/PR9.0000000000000843

**Published:** 2020-09-23

**Authors:** Alice Kongsted, Peter Kent, Jonathan G. Quicke, Søren T. Skou, Jonathan C. Hill

**Affiliations:** a Department of Sports Science and Clinical Biomechanics, University of Southern Denmark, Odense M, Denmark; b Nordic Institute of Chiropractic and Clinical Biomechanics, Odense, Denmark; c School of Physiotherapy and Exercise Science, Curtin University, Perth, Australia; d Primary Care Centre Versus Arthritis, Research Institute of Primary Care and Health Sciences, Keele University, Staffordshire, United Kingdom; e Department of Physiotherapy and Occupational Therapy, Næstved-Slagelse-Ringsted Hospitals, Slagelse, Denmark

**Keywords:** Models of care, Osteoarthritis, Low back pain, Risk-stratified care, Stepped care, Decision making

## Abstract

The overall quality of care for musculoskeletal pain conditions is suboptimal, partly due to a considerable evidence-practice gap. In osteoarthritis and low back pain, structured models of care exist to help overcome that challenge. In osteoarthritis, focus is on stepped care models, where treatment decisions are guided by response to treatment, and increasingly comprehensive interventions are only offered to people with inadequate response to more simple care. In low back pain, the most widely known approach is based on risk stratification, where patients with higher predicted risk of poor outcome are offered more comprehensive care. For both conditions, the recommended interventions and models of care share many commonalities and there is no evidence that one model of care is more effective than the other. Limitations of existing models of care include a lack of integrated information on social factors, comorbid conditions, and previous treatment experience, and they do not support an interplay between health care, self-management, and community-based activities. Moving forwards, a common model across musculoskeletal conditions seems realistic, which points to an opportunity for reducing the complexity of implementation. We foresee this development will use big data sources and machine-learning methods to combine stepped and risk-stratified care and to integrate self-management support and patient-centred care to a greater extent in future models of care.

## Abstract 2

Supplemental Digital Content is Available in the Text.

Substantial overlap between interventions and models of care for osteoarthritis and low back pain suggests potential for one common model, which may facilitate implementation.

## 1. Background

Musculoskeletal pain conditions are the largest causes of disability worldwide.^30^ Among the most disabling are knee and hip osteoarthritis (OA) and low back pain (LBP), which affect approximately 303 and 577 million people, respectively, resulting in very large annual societal costs.^30,35,86^ An important contributor to these costs is poor-quality health care, including the application of non–evidence-based treatments. Examples of this are the overuse of imaging, surgery, and opioids, in circumstances when this is not aligned with current evidence-based recommendations for first-line care.^8,12,27,98,99^

This suboptimal quality of care is in part due to the poor implementation of evidence-based guidelines for both OA and LBP.^24,27,32,37,62,88,94^ Bridging this evidence gap to provide effective and affordable health care while ceasing the use of harmful, non–guideline-adherent practices is a major challenge.^12,24,27,31^ However, it has great potential to significantly improve health and reduce costs.^12,26^

Models of care that support best practice clinical decision-making are potentially very useful for implementing guideline-recommended care options (Table 1 for definitions of key terms).^10,25^ In OA and LBP, stepped care and stratified care models have been suggested to support decisions about care. With stepped care, all patients are initially offered the same basic care, and subsequent treatment efforts are only increased if patients have not benefited sufficiently. An example of a stepped care model is the “Beating Osteoarthritis” (BART) model.^95^ Similarly, the “Good Life with osteoArthritis in Denmark (GLA:D)” program is considered the initial level in a stepped approach^92^ (Appendix A: Summary of example models; available as supplemental digital content at http://links.lww.com/PR9/A76). In a risk-stratified care model, treatment is targeted to patient subgroups when patients initially seek care, with more comprehensive care offered to patients at risk of poor outcomes. The most widely known example of this approach is the STarT Back Screening Tool with treatments matched to risk profiles of people with LBP.^44,96^

**Table 1:** Definitions of key terms as used in this article.

Term	Definition
Clinical guidelines	Recommendations for clinical practice informed by systematic reviews of evidence and expert opinion, including an assessment of the benefits and harms of alternative care options.
Model of care	Framework that describes the principles of disease‐specific, evidence‐informed health care that should be delivered to consumers in a given setting, that is, the right care, at the right time, delivered by the right team, in the right place, using the right resources^10^
Moderator	A factor (or combination of factors) that affects the direction and/or strength of the relationship between the exposure (eg, treatment) and the outcome. For example, a factor that affects the response to a treatment.
Prediction model/tool	A model/tool consisting of factors that in combination help predict the likely outcome of an individual with a given intervention
Treatment decision tool	A tool consisting of factors that in combination helps predict the likely effect of one treatment compared with another treatment
Risk stratification tool	A tool that combines a prediction model (of risk of poor outcome) with treatment options for each risk stratum

To engage with the international challenges around improving the management of musculoskeletal health conditions, there is a need for models of care that can be widely implemented. With recent reviews highlighting several similarities between recommended treatment options and clinical practice guidelines for OA, LBP, and other musculoskeletal conditions, it seems that similar models of care might also be appropriate, which would reduce the complexity of implementation.^5,58^ Exploring the generalisability of models of care across conditions would inform clinicians, researchers, and decision-makers about ways of developing models of care and may facilitate implementation in clinical practice.

Therefore, the aim of this overview was to examine the conceptual similarities and differences between stepped care and risk-stratified care, discuss lessons learned from work on these models, and describe possible future directions for the field.

## 2. Key message #1: models of care for osteoarthritis and low back pain share many similarities

Multiple national and international guidelines exist for the care and management of OA and LBP.^58^ These clinical guidelines typically offer a series of recommendations for clinical procedures and treatments with some offering explicit or implicit recommendations about models of care including stratified care models, stepped care models, and hybrids of the 2. For example, the NICE guidelines from the United Kingdom explicitly recommend risk-stratified care for LBP,^68^ whereas the OARSI guidelines for knee and hip OA recommend a stepped care approach including a core initial treatment for all patients consisting of education and exercise with or without weight management.^8^ Furthermore, the OARSI guidelines advocate elements of stratified care by differentiating care for people with and without comorbidities.

Examples of guidelines that implicitly recommend stepped care are the recommendations for OA and LBP management stating that surgery is only considered for specific groups of patients and only if nonsurgical care of a sufficient dose has already been provided without adequate symptom relief.^8,58,73^ Another example is the recommendation that pharmacological treatment is only initiated for chronic LBP in patients who had inadequate response to nonpharmacological therapy.^79^

In OA, stepped care is the most commonly advocated model of care, whereas in LBP hybrid models, elements of both stepped and risk-stratified care are included in clinical guidelines.^8,73^ Both models have basic treatments (recommended for everybody with the condition), adjunct treatments (for some), and surgical options (for a minority).^8,44,73^ Despite some differences in models of care, many aspects of treatment essentially remain the same. For example, self-management advice and education are included for all patients, and should be continuously delivered throughout all steps of the treatment pathway.^8,68,73^

Exercise therapy is also a core element in the treatment of both OA and LBP due to its therapeutic effects in these conditions, the concurrent benefits of physical activity for common comorbidities and general health, and because it is a safe treatment option.^9,75,80,91^ Examples in OA are the Enabling Self-management and Coping with Arthritis Knee Pain through Exercise (ESCAPE-knee pain) and Beating Osteoarthritis (BART) programs in the United Kingdom, the PARTNER model in Australia, and GLA:D for knee and hip offered in several countries in the world.^3,47,48,84,92^ These are all stepped care models that include supervised exercises as part of the treatment, either for all patients (ESCAPE, PARTNER, and GLA:D) or where self-management advice has proven insufficient (BART). In LBP, most clinical guidelines do not recommend supervised exercises for acute LBP, but consistently do so for persistent LBP.^4,73^ Following the stratified care principle, the NICE guidelines recommend that the decision about exercise therapy is guided by the STarT Back model for LBP with supervised exercises provided for people at medium risk and high risk of having persistent activity limitation after 6 months, irrespective of episode duration.^68^

Adjunct and more intensive treatments, such as psychologically informed physiotherapy using a combined physical and psychological approach, are perhaps where the greatest differences between OA and LBP models are presently seen. Some LBP guidelines stress the importance of early identification of those at high risk of poor clinical outcome due to psychosocial obstacles to recovery in order that they are fast-tracked to therapists who can address these issues,^68^ whereas OA guidelines have until recently only included psychological treatments to a lesser extent.^67^ However, recent OA guidelines have recommended addressing psychological factors in certain patient subgroups and are, in this aspect, suggesting a stratified approach.^8^ Also, return-to-work interventions are recommended without delay in both OA and LBP for subgroups who are struggling with their capacity to work or have been absent from work. Differences in treatment recommendations include that weight loss, orthoses, and corticosteroid injections can play a role in the treatment of OA, whereas that is not the case in LBP. Best practice for pain medication also differs to some extent for OA and LBP, with stepped models in OA suggesting different tiers of medication be tried in a stepped fashion, whereas in LBP, the role of pain medication is being questioned because of lack of effect and risk of harm.^87^

Finally, decision-making about referral for surgical opinion is similar for OA and LBP in the sense that surgery is only considered if best-practice nonsurgical treatments have not provided sufficiently good outcomes. However, currently, there is a lack of evidence for surgical treatment as an effective option in nonspecific LBP.^27,73,97^

In summary, clinical guidelines for both OA and LBP recommend elements of stepped care and stratified care models, including some explicit recommendations of stepped care in OA and stratified care in LBP. Although the same core treatments are recommended for patients with OA and LBP, some differences emerge in the clinical decision-making process around some adjunct therapies.

## 3. Key message #2: it is not a choice between stepped care or stratified care—rather, it is about using the best from both of them

A fundamental difference between these 2 models of care is that stepped care assumes a substantial number of patients will improve with core treatment, and patients who do not are not harmed by waiting for more comprehensive treatment to be initiated.^59^ By contrast, risk-stratified care assumes that patients with poor outcomes can be identified at an early point of care-seeking and their risk of poor outcomes reduced by early targeted interventions.^45,59^

The risk of overtreatment and undertreatment with stepped care clearly depends on the content of care at each step. If the initial step only includes, eg, self-management advice, more people are potentially being undertreated with a stepped approach than by risk stratification. By contrast, where more comprehensive core treatment packages are the initial level of care (eg, including pain medication), there is a risk of overtreatment and adverse events in patients who would improve sufficiently with self-management advice alone. The likelihood that patients are overtreated or undertreated with risk-stratified care depends on the accuracy with which patients benefitting from more intensive care can be identified.^33,51^ In addition to an accurate screening tool, better outcomes rely on there being suitably effective treatment options available for each risk stratum. Both models of care guide which general approach to follow, with room for clinical judgement and individualisation of care that directs treatment at patients' individual prognostic factors, impairments, and treatment preferences.

Despite differences, the fundamental approach that informs both of these models is that treatment choice differs between patients based on individual patient information. In stepped care, this information is the response to prior treatment and in risk-stratified care, it is information on prognostic factors (the predicted outcome).^36,72^ Consequently, patients seeking care for the first time with an estimated good prognosis will have the same treatment with stepped or stratified care. This may also apply to patients seeking care early after symptom onset because there is greater uncertainty with prediction in the acute stage.^63^

To the best of our knowledge, no trials have directly compared the effectiveness of different models of care in musculoskeletal conditions. Stratified care for LBP based on the STarT Back Screening Tool was cost-effective when compared with usual care (no specified model of care) in a U.K. effectiveness trial and in an implementation study, with usual care being determined by the individualised treatment decisions made by general practitioners.^28,44^ In an Irish nonrandomised controlled study, improved outcomes were observed for high-risk patients with risk stratification, seemingly without affecting outcomes for low-risk and medium-risk patients.^66^ Also, an implementation strategy, including the use of the STarT Back Screening Tool in Danish general practice, led to lower rates of referral to hospital settings.^83^ However, the implementation of the STarT model was not as successful in a U.S. study,^15^ illustrating that effective implementation strategies for models of care may vary across different international health service contexts. The effectiveness of a stepped care model for other musculoskeletal conditions has not been investigated using a controlled design. However, a randomised controlled trial in knee OA has demonstrated that a substantial proportion of patients considered eligible for surgery achieved satisfactory outcomes with alternative nonsurgical treatment and decided not to proceed with surgery.^93^ This implied that the stepped approach of offering core nonsurgical treatments before considering surgery is an effective strategy for some patients.

Currently, there are a number of trials investigating the effectiveness of risk-stratified care for LBP in different settings and countries,^13,38,64^ and trials are being conducted to test stepped care approaches in OA^2^ and LBP^81^ (examples were identified from ClinicalTrials.gov). Also, we identified one published pilot study and protocol for a trial testing stratified care in musculoskeletal conditions including OA.^39,40^ No trials or protocols were identified on head-to-head comparisons of stepped and stratified models of care. Although there is limited research to inform whether models of care could be the same for OA and LBP or if one model should be chosen rather than the other, a recent focus group study indicated that practitioners consider a generic stratified model to be relevant across 5 musculoskeletal conditions.^78^

**Figure 1.**
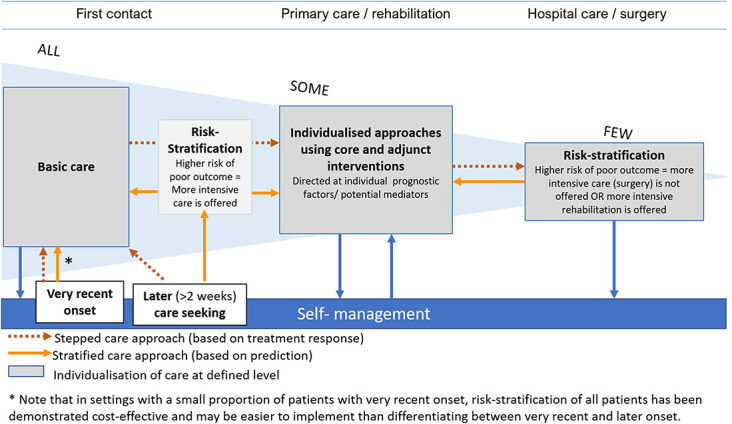
Principles of combining stepped and stratified models of care. Existing models of care coexist in musculoskeletal care to form a joint model with stepped and stratified approaches to making treatment decisions being used at different points along the clinical course. The figure illustrates principles and some parts of this combined model remain to be investigated.

Given the similarities and overlapping practical applications of stepped and risk-stratified care, these models may be considered parts of a common approach with elements of each model being present at different time points in decision-making (Fig. 1). As described in key message #1, such a combined approach is implicitly advocated by current clinical guidelines. A stepped approach is used in the very early stage of symptoms, where all patients are offered basic care supporting self-management.^2,20,97^ From primary care settings, which mostly managed nonacute LBP presentations, there is evidence that risk stratification can be cost-effective as a tool for making decisions about care pathways. The precise content of treatment is to some extent individualised within both stepped and stratified care. For example, in the GLA:D programs for knee, hip, and back pain and the ESCAPE-knee pain intervention, there is individualisation of exercise programs by adaptation of type and dose to the individual patient.^48,92^ Similarly, in LBP, patients who are stratified to the same level according to the STarT Back Screening Tool have different treatments tailored to their individual risk factors.^36,92^ In both OA and LBP, decisions about surgical care follow a stepped approach based on observed response to nonsurgical care (except for very special cases of trauma or progressive neurological deficits).^8,50^ At the level of surgical assessments, risk stratification again has a role, which may mean that people with a high risk of poor outcome from surgery would not be recommended this treatment or are perhaps offered more intensive rehabilitation (Fig. 1). However, although there are known risk factors for poor outcome with surgery, currently there are no well-established decision support tools for surgery or other secondary care settings. There is a model for predicting outcome of total knee arthroplasty albeit with only partial support for its external validity.^21,82^ Also, a decision support tool for spinal fusion surgery is being developed,^85^ but there is no evidence yet that implementation of risk stratification on the basis of these 2 approaches does actually improve patient outcomes. There is evidence that the STarT Back Screening Tool, which was developed for primary care, is not useful for predicting outcomes in secondary care settings,^52,65^ but stratified care for LBP (matched treatment to risk profiles) has not been investigated in secondary care settings. Also, at this point, there is no evidence to inform exactly how models of care might most optimally be combined.

Although musculoskeletal conditions are very often recurrent,^18,19,71,74^ and many people with OA and LBP seek care repeatedly with periods of self-management in between, none of the existing models explicitly suggest how recurrence is handled. Episodic pain generally has less negative impact than persistent pain and recurrences do not necessarily indicate progression of the condition.^55^ Therefore, recurrences should not automatically lead to more intensive care, and treatment decisions should rationally take previous treatment response and success with self-management into account.

## 4. Key message #3: risk stratification has different roles and implications depending on how, when, and where it is applied

In general, within risk stratification tools, there are 2 different types of prognostic factors: those that are treatment-modifiable (eg, pain, fear, and pain catastrophising) and those that cannot be modified with treatment (eg, number of previous recurrences, age, and previous surgery). This distinction becomes important when designing prognostic tools and prediction models because both types of prognostic factors have different roles and implications for clinical practice. The inclusion of treatment modifiable factors has the benefit that it gives clinicians some signals about potential treatment targets. By contrast, nonmodifiable factors, although they do offer useful prognostic information, are not helpful for identifying the specific target of treatments but could have the advantage, by their very nature, of being more stable than modifiable factors in their predictive abilities across different settings. An example to illustrate that prediction by modifiable factors can be unstable is the finding that the STarT Back Screening Tool (which only includes modifiable factors) had poor predictive performance in emergency care^61^ and among patients with a very short LBP duration (<2 weeks) in primary care.^63^ In both settings, this could be because many of the factors screened (eg, catastrophising, anxiety, and fear) are not sufficiently stable in very acute patients to be reliable prognostic indicators in these situations. The inclusion of some nonmodifiable factors in a predictive tool designed for the fast-changing emergency context is, therefore, being planned as a possible way of adding greater predictive stability to risk tools in this setting. Prognostic factors that are not modifiable by treatment, eg, socioeconomic status, also play an important role in identifying people unlikely to benefit from standard interventions, who should be offered support that takes their life circumstances into account.^22^

Another important issue for the development and application of prognostic models as part of decision-making is the need to be clear about the exact outcome the model is designed to predict and the exact purpose of using the model. In principle, there are 3 different purposes of prediction in models of care: (1) Estimating prognosis with a given treatment or in a given setting regardless of potential treatment differences (what is the likely outcome of this patient with this treatment/in this setting?); (2) Informing the extent/intensity of care (which care pathway is best for this patient considering both benefits and harms?); and (3) Informing the type of treatment (which treatment is most effective for this patient?) (Table 2).

**Table 2:** Purposes and principles of prognostic models in decision-making.

Purpose	Example* (level of evaluation)	Principle	Effectiveness testing	Proposed term
To inform expected prognosis, given a specific treatment or care pathway *“What is the likely outcome of this patient with this treatment/in this setting?”*	Predicting number of days to recovery (pain ≤1 on a 0–10 scale) in people with acute LBP visiting primary care “The Hancock model” (externally validated; no test of impact)^17,34,101^	Patients predicted to have a good outcome have minimal intervention, with more attention paid to those predicted to have poor outcome. No recommended matched treatments detailed and the influence of care on the outcome is not considered.	Effectiveness is tested in studies investigating if use of the prediction model affects, eg, patient outcomes, care pathways, or costs. This may be in a “subgroup system RCT”^53^	Prediction model/rule/tool
To guide which care pathway to take/level of care to provide *“Which care pathway is best for this patient?”*	Determining care pathway based on predicted risk of activity limitation after 6 months at the initial point of care-seeking. “The STarT back screening tool” (externally validated effectiveness trial, impact study, mediation analyses to confirm treatment targets)^28,41,44,60^	Patients in subgroups at increased risk of poor prognosis are offered more comprehensive care. Recommended matched treatments for risk subgroups are based on expert opinion.	Effectiveness is tested in trials comparing the risk-stratified model of care to usual nonstratified care or an alternative model of care. This is referred to as a “subgroup system RCT”^53^	Stratified care tool
To guide whether to provide a specific type of treatment *“Which treatment is likely to be most effective for this patient?*”	Deciding treatment choice based on predicted outcome with one specific treatment compared with another treatment (no validated examples from OA or LBP)	Patients positive on factors associated with better outcome with treatment A than with treatment B (“treatment effect moderators”) are offered treatment A.	Treatment decision tools are both designed and tested in a “2-group plus subgroup RCT”^53^	Treatment decision rule/tool

* Examples of the purpose. There is no evidence of effectiveness/clinical impact of all types.

LBP, low back pain; OA, osteoarthritis; RCT, randomised controlled trial.

Those distinctions are important because the research methods used to develop and validate different types of models are completely different. Tools meant to predict outcome in a given setting are based on prognostic models developed using observational cohort data, whereas tools designed to inform decisions about care pathways or treatments typically require moderators of treatment effect to be identified using data from randomised clinical trials.^42^ An alternative method is to conduct a randomised controlled trial to compare the combined effect of using a prognostic model developed from a cohort study and matched care pathways/interventions against another approach (eg, usual nonstratified care). However, this has some limitations because it is not able to confirm (without the trial being powered to test for a treatment interaction effect) whether the matched treatments used are indeed moderators of treatment effect. All types of prognostic models need external validation and the testing of clinical implications of using the tool before implementation.^16^

The subtle but important differences between types of prognostic models are the source of much confusion, particularly because the clinical implications from the types of tools are very different. In the case of a prediction tool designed to predict a patient's likely response to a specific treatment, patients who are classified as “high risk” means they are at high risk of treatment failure, and so the clinical implication is to avoid giving that patient that specific treatment. However, with risk stratification tools, there are different treatment options suggested for each of the risk strata (subgroups at increasing risk of poor outcome), and patients who are classified as “high risk” in this context typically receive a greater intensity of treatment and/or more complex treatment. It is therefore easy to see why clinicians are often confused by the term “high risk” because the term means very different things depending on the type of predictive tool being applied.

Finally, as mentioned under key message #2, there is a need to consider a person's long-term history and previous episodes when models of care are applied, which existing risk stratification tools currently do not measure. For example, patients might be classified as being at a “low risk” of persistent disability, but they are actually troubled by repeated episodes that cumulatively have a considerable impact on their life. To overcome this issue, we anticipate that future risk stratification tools will likely need to include trajectory information to better inform a longer-term, rather than short-term, treatment perspective. For example, they might include information from the validated self-reported Visual Trajectories Questionnaire (VTQ-Pain) that asks patients to choose from 8 picture diagrams to identify their longer-term pain experience,^23^ or use automated searches of electronic medical record data to capture an individual's medical history and treatment experience.

## 5. Key message #4: future directions

In the future, models of care for OA and LBP are likely to include: (1) greater precision of risk assessment and individualised treatment through better technologies, (2) greater patient agency through self-care/community-based care and digital health awareness, and (3) more advanced training of primary care clinicians to use digital health tools and to engage in the psychosocial aspects of musculoskeletal conditions.

### 5.1. Better technology

Advances in technology have the potential to integrate stratified, stepped, and individualised care with the common aim of identifying the “the right treatment for the right patient at the right time.” One aspect of this is the development of dynamic prediction models based on machine learning (artificial intelligence) instead of developing a single prediction model based on a sample of the population at a single point in time. Such models are dynamically updated in response to new people entering the sample, prognostic variables being refined over time, and changes in prognosis due to the setting, or advances in treatments and models of care.^49^ Another advantage is the capacity to update prognostic estimates for individuals over time by using clinical course data as they emerge, thereby better mimicking clinical reasoning than traditional approaches to prediction modelling.^100^ So, dynamically updated models have the capacity to include elements of both stepped care (based on response to care) and stratified care (based on prognostic factors).

Other technical advances are the integration of synergistic technologies to improve models by the inclusion of data from multiple sources, such as large (big data) clinical databases, wearable sensors, and mobile device apps. The Back-Up Project^7^ and the Self-Back Project^89^ are examples of projects that integrate these technologies in clinical decision tools for LBP in first-contact settings and ongoing care settings and also for self-management. An example of using information from wearable sensors is the use of real-time feedback on back position and movement in the treatment of LBP.^54^

The use of these technologies has the potential to facilitate the regular follow-up and monitoring of chronic health conditions, such as knee OA and LBP, where self-management including exercise is a core component. However, barriers exist to the development of these integrated technology approaches, including cost, clinician time constraints and cultural resistance, and concerns about the privacy of patient data.^6^ As with any sociotechnical system, the most successful approaches will require a combination of tangible health benefits, timely and patient-enabling information, user-friendly and accessible technology, and affordability.

More broadly, there is an emerging hybrid model of care with a flexible mix of both face-to-face health care and digital health solutions depending on the individual's needs. Better technologies are increasingly providing enabling information during assessment, diagnosis and treatment decision-making, and the automation of components for monitoring patient progress.

### 5.2. Advanced training of primary care clinicians

In recognition of the multidimensionality of persistent musculoskeletal conditions,^29^ risk stratification and targeted care increasingly traverse biopsychosocial factors. Because patient and clinician cognitions and emotions about health conditions are strongly prognostic,^76^ there is considerable research being conducted into a range of targeted treatment approaches that include screening for, and engagement with, these psychosocial aspects when appropriate.^90^ However, there are a number of challenges to be addressed, including primary care clinician time constraints, clinician professional self-image and perceptions of scope of practice,^1,77^ and a lack of clarity about the best models of training, and for whom they are most appropriate.

As digital health technologies to assist diagnosis, support treatment, report patient outcomes and experiences increasingly merge and blend into comprehensive tools, this will have an impact on clinician training needs. For example, new digital health technologies are already being developed to support back pain clinical decision-making, such as the Back-UP project,^7^ which is digitising risk stratification and the monitoring of patient outcomes during rehabilitation. It is also likely that existing clinical decision support tools for OA, such as the Arthritis Alliance of Canada's OA Tool (https://www.cfpc.ca/uploadedFiles/CPD/OATOOL_FINAL_Sept14_ENG.pdf), will be transferred from existing paper-based formats into a digital health technology. It is likely that these types of tools will increasingly be integrated into existing medical health record and patient management systems and that process will simultaneously require clinician training to understand how to best make use of these technologies and the metrics they provide. We expect this change to be driven by both the potential of these technologies to facilitate improvements in the quality of care and by their ability to help secure better informed funding of clinical services.

### 5.3. Greater patient agency

There is an increasing recognition of the merit of demedicalising musculoskeletal care and shifting towards models of care that are more patient-centred and focused on supporting self-management.^12,57^ There is also a shift away from defining health as “complete well-being” to defining health as “an ability to adapt and self-manage” and towards models of care that integrate health care with self-care and community-based activities.^46^ For example, although only a minority of people with OA regularly engage in physical activity at recommended levels, a clinical trial in Canada showed that both community-based and home-based walking programs can be effective strategies to manage mild to moderate OA of the knee.^11^ Quality of life and clinical outcomes improved among participants across a 12-month intervention period and continued to improve 6 months later.

The management of chronic conditions is increasingly about supporting patients' decisions as well as clinicians' decisions, which should be reflected in decision support tools for patients and clinicians. Decision support tools aimed at patients will have to take individual capacities, other health conditions, and social context into account. Future models of care will also need to address the social inequalities in health that are not necessarily captured by illness-specific or modifiable risk factors.^14^ Greater patient agency is also being promoted through trusted review websites (eg, www.orcha.co.uk) that allow end-users to compare different digital health products, such as patient apps and health websites.

The clinical utility of these 3 emerging and enabling directions (greater precision through better technologies, greater patient agency, and more advanced training of primary care clinicians) will remain contingent on appropriate evaluations of benefits, harms, costs, and their potential for implementation, for example, using guidance from the Evidence Standards Framework for Digital Health Technologies published by NICE.^69^

## 6. Discussion

Models of care provide a framework for implementing evidence into practice and reducing the variation in care. At present, there is no direct evidence that one particular care model is superior to another, and the approaches of stepped and stratified care both play a role in the organisation of interventions for OA and LBP. This overview illustrated that the content of care and the overall approach to treatment decisions share many similarities in both OA and LBP, and a common model of care emerged. In both OA and LBP, all patients should receive information about their condition and advice on self-management, and also supervised exercises are part of core treatment in both OA and persistent LBP, with other optional interventions for some patients. Currently, risk stratification is only applied in LBP, but whether effectiveness of care can be improved by applying risk stratification in other musculoskeletal conditions is also being investigated.^43,70^ Generally, the observed differences in the models of care for OA and LBP are not substantiated by evidence, and the potential for integrated models across musculoskeletal conditions deserves attention.

In the development, testing, and implementation of models of care, there are some potential pitfalls to be aware of. Importantly, careful consideration should be given to whether a decision support tool is meant for predicting outcome irrespective of treatment (to decide the level of attention needed), for predicting likely outcome given a specific treatment (to decide if that specific intervention should be offered or not), or for predicting added benefit from one treatment compared with another (to decide which treatment to choose).

Limitations of current models include that they do not sufficiently guide clinicians in integrating information about patients' symptom trajectory, nonmodifiable prognostic factors, comorbid conditions, and previous treatment response, and they do not support an interplay between health care, self-management, and community-based activities. Also, the way in which models of care might best be adapted to differences in the local health care contexts is still unknown. We foresee that future models of care will use big data sources and machine-learning methods to overcome some of these limitations. Even without the use of new technologies, models of care for musculoskeletal conditions need development to include existing knowledge of symptom trajectories,^56^ and to integrate self-management support and patient-centred care to a larger extent.^8,12^

In summary, existing models of care for OA and LBP show good potential for improving management of musculoskeletal health care and they share many commonalities. This points to an opportunity for reducing complexity of implementation of guideline-recommended interventions by standardisation across health conditions. Nonetheless, individual care decisions are complex, and implementation strategies will differ across healthcare contexts. We foresee that insights from existing and future research will combine with new technologies to overcome elements of this complexity with promise for better models of care.

## Disclosures

The authors declare there are no conflicts of interest.

A. Kongsted's position at the University of Southern Denmark is partly financed by an unrestricted grant from the Foundation for Chiropractic Research and Postgraduate Education. J.C. Hill, S.T. Skou, and A. Kongsted are, respectively, part of the groups that developed the STarT Back Screening Tool, the GLA:D program for knee/hip, and the GLA:D program for LBP. A. Kongsted is an associate editor of the BMC Chiropractic and Manual Therapy journal. S.T. Skou is an associate editor of the Journal of Orthopaedic & Sports Physical Therapy, and has received grants from The Lundbeck Foundation and personal fees from Munksgaard, all of which are outside the submitted work. S.T. Skou is currently funded by grants from the European Research Council (ERC) under the European Union's Horizon 2020 research and innovation program (grant agreement No 801790) and from Region Zealand (Exercise First), both unrelated to the current study. J.G. Quicke is partly funded by a NIHR Clinical Research Network West Midlands, Research Scholar Fellowship, and is a member of the NICE OA guideline committee. The views expressed in this publication are those of the authors and not necessarily those of the National Health Service, the National Institute for Health Research, or the Department of Health and Social Care, or NICE.

## Appendix A. Supplemental digital content

Supplemental digital content associated with this article can be found online at http://links.lww.com/PR9/A76.
